# Stereoselective Synthesis of β-Glycinamide Ribonucleotide

**DOI:** 10.3390/molecules27082528

**Published:** 2022-04-14

**Authors:** Lisa Ngu, Debarpita Ray, Samantha S. Watson, Penny J. Beuning, Mary Jo Ondrechen, George A. O’Doherty

**Affiliations:** Department of Chemistry, Northeastern University, Boston, MA 02115, USA; ngu.l@husky.neu.edu (L.N.); debarpita.ray@gmail.com (D.R.); watson.sa@husky.neu.edu (S.S.W.)

**Keywords:** β-glycinamide ribonucleotide, β-GAR, GART, stereoselective synthesis

## Abstract

A diastereoselective synthesis of the β-anomer of glycinamide ribonucleotide (β-GAR) has been developed. The synthesis was accomplished in nine steps from D-ribose and occurred in 5% overall yield. The route provided material on the multi-milligram scale. The synthetic β-GAR formed was remarkably resistant to anomerization both in solution and as a solid.

## 1. Introduction

**Scheme 1:**
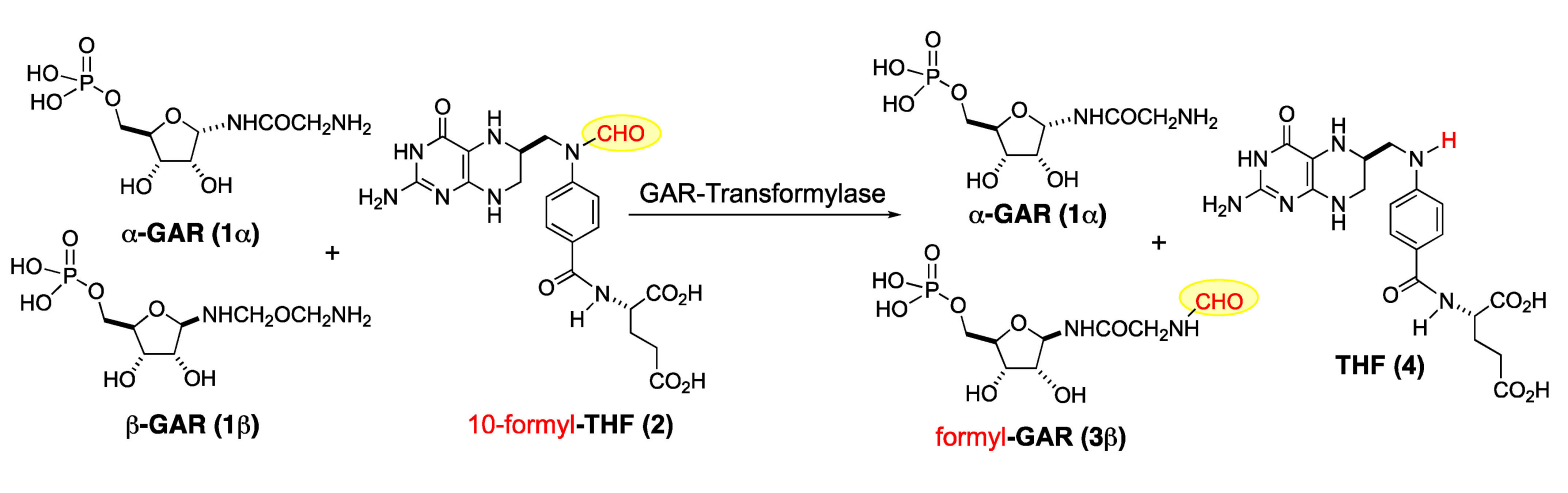
GART mediated transfer of formyl group from 10-formyl-THF to GAR.

The beta anomer of the glycinamide ribonucleotide, also known as β-GAR, is an intermediate in the biosynthesis of the nucleic acid building blocks of DNA. The formylation of glycinamide ribonucleotide (β-GAR) to form *N*-formylglycinamide ribonucleotide (fGAR) is catalyzed by the enzyme glycinamide ribonucleotide transformylase (GART). The enzyme reaction catalyzed by GART transfers a formyl group to β-GAR from the 10-*N*-postion in 10-formyl-tetrahydrofolic acid (10-formyl-THF) to form tetrahydrofolic acid (THF) and fGAR. The formylation of β-GAR is an important step in the de novo purine biosynthesis pathway [1,2,3]. Thus, the selective inhibition of the *N*-formylation of β-GAR (e.g., in humans or bacteria) holds therapeutic potential [4,5,6]. In this context, a GART assay was developed that uses formyldideazafolic acid (fDDF) as a substitute for 10-formyl-THF [7,8]. Importantly, it has been shown that this assay can be conducted using a 1:1 mixture of GAR anomer and α- and β-GAR (Scheme 1) [3,9,10,11,12]. Out of an abundance of caution, we desired the ability to run this assay with both diastereomerically pure β-GAR and the mixture of diastereomers (α-/β-GAR) [13].

**Scheme 2:**
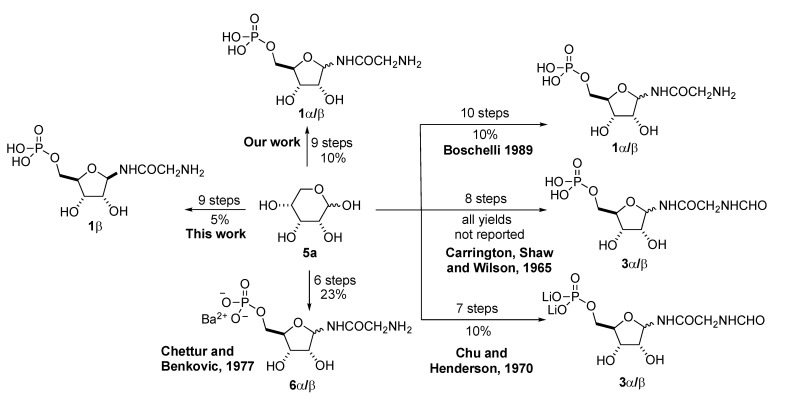
Previous approaches to GAR.

Previously, we published a practical synthesis of α-/β-GAR (**1**-α/β) [14]. In addition to our report, others have disclosed syntheses of the anomeric mixture α-/β-GAR (**1**-α/β). These efforts are outlined in Scheme 2 [15,16,17,18]. To the best of our knowledge, current access to anomerically pure β-GAR comes from its enzymatic synthesis or chromatographic separation of the anomers using reverse phase methods. In our hands, both these methods have their limitations, when it comes to providing material at ~100 mg scale for further studies. Herein, we disclose a practical stereoselective synthesis of β-GAR and report upon its anomeric stability. This route to β-GAR builds upon our previous experience with the synthesis of α-/β-GAR (**1**-α/β), which in turn rests on the earlier work of Boschelli [17] and Chu and Henderson [18] (Scheme 2).

**Scheme 3:**
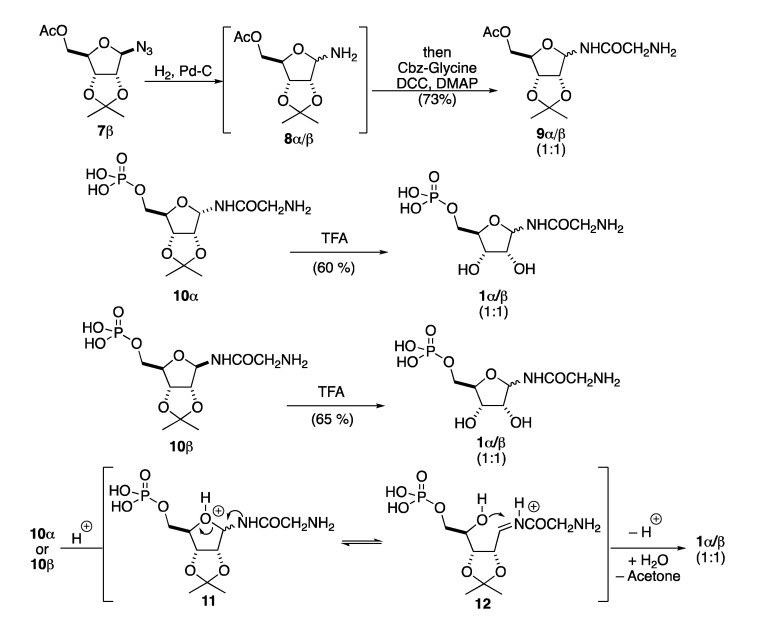
Previous issues regarding anomeric stereochemistry in β-GAR synthesis.

A guiding principle that informed our approach is the difficult handling of the zwitterionic amino-phosphate β-GAR, which is both very polar and water soluble. In addition, from our previous efforts, we were aware of the stereochemical instability of the anomeric position during the acylation of the glycosidic amine and the acetonide deprotection (Scheme 3). More specifically, in our previous synthesis, we were not able to maintain the β-anomeric stereochemistry during the reduction of the azide **7**β and the acylation of the resulting aminoglycoside **8**α/β to form **9**α/β. This was presumably due to faster anomerization of **8**α/β than the corresponding acylation reaction. Similarly, we were not able to find mild enough conditions for the selective acid-catalyzed removal of the acetonide group in **10**α or **10**β to form **1**α or **1**β without rapid acid-catalyzed anomerization. We hypothesized that this anomerization occurred through protonation of the ring oxygen in **10** to form **11** followed by a ring opening/closing anomerization mechanism (i.e., **11** to **12**), before acetonide removal (i.e., **12** to **1**α/β).

## 2. Results

**Scheme 4:**
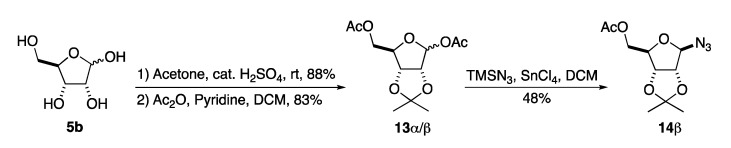
Synthesis of D-ribofuranosyl azide (**3**).

To address the issue with anomeric instability we decided to change the *C*-2/3 acetonide protecting group to a benzylidene acetal. This switch had the advantage of allowing for a neutral final deprotection via hydrogenolysis. In addition, we hoped the benzylidine acetal would allow for improved selectivity in the azide reduction/acylation step. We have previously found in several aminosugar syntheses that late state per-hydrogenolysis allows for minimal purification of highly polar compounds [19,20,21,22,23,24,25,26,27]. Our route to β-GAR started with **14**β, which we previously prepared in our synthesis of α-/β-GAR (**1**α/β) (Scheme 4). The synthesis of **14**β began with an acid catalyzed acetonide protection of D-ribose **5b** to form the *C*-2/3-acetonide and acylation of the *C*-1 and *C*-5 position to form **13**α/β. Then, a tin tetrachloride promoted azide displacement of the anomeric acetate gave azide **14**β in acceptable overall yield (35%) and excellent stereoselectivity.

**Scheme 5:**
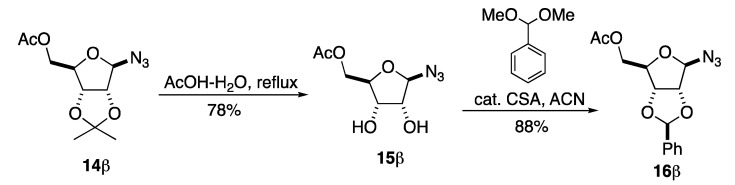
Switching from isopropylidene to benzylidene protected ribofuranosyl azide (**5**).

The acetonide on **14**β was readily replaced by a two-step process (Scheme 5). This began with an acetonide deprotection with refluxing acetic acid to give diol **15**β without any sign of anomerization. Then, the diol of **15**β was stereoselectively protected as a benzylidene acetal with dimethoxy toluene in the presence of a catalytic amount of (+/−)-camphor sulfonic acid (CSA) to give azide **16**β in good yield and with excellent stereoselectivity. The choice of chiral racemic CSA as the catalytic acid for the benzylidene formation was based on its generally improved solubility in organic solvents over *p*-TsOH.

**Scheme 6:**
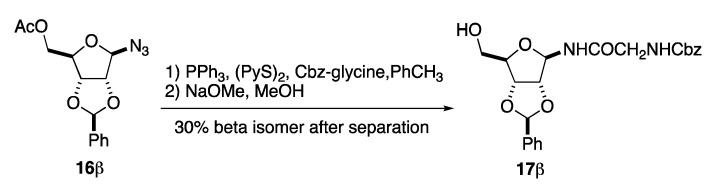
Synthesis and separation of Cbz-Glycinamide of benzylidene protected-d-ribose.

Next, we looked into the selective reduction of the anomeric azide in **16**β in the presence of a *N*-Cbz-glycine acylating agent (Scheme 6). We found PPh_3_ to adequately accomplish the selective reduction. However, finding an acylating agent that matched these conditions was more difficult. After much experimentation, we found that this was most successfully accomplished by exposing anomeric azide **16**β to a 1:1:1 tertiary mixture of PPh_3_, (PyS)_2_, and *N*-Cbz-glycine in toluene [28]. After passing through a plug of silica gel, the *C*-5 acetate on the product was selectively hydrolyzed in basic MeOH to give the anomeric glycine amide **17**β, albeit in a less than ideal yield (30%). Unfortunately, the issue with the slowly reacting anomeric amine led to a significant amount of methanolysis of the anomeric amine product. Fortunately, the desired β-GAR precursor **17**β could be isolated as a single diastereomer.

**Scheme 7:**
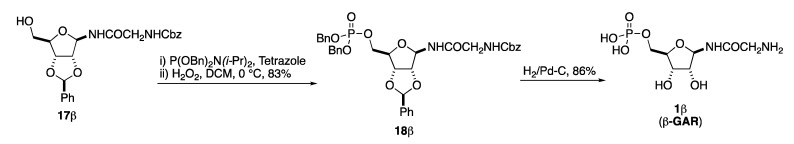
Installation of phosphate and deprotection.

Finally, all that remained was to install the *C*-5 phosphate and deprotect the benzyl protecting groups (Scheme 7). This began with the introduction on **17**β of a dibenzylphosphite group with (BnO)_2_PN*i*-Pr_2_ and tetrazole, followed by a phosphite to phosphate oxidation with hydrogen peroxide to give **18**β in an 83% overall yield [14,17]. Finally, the synthesis of β-GAR (**1**β) was completed by a global deprotection via a per-hydrogenolysis. Thus, the two benzyl phosphate groups, the benzylidene acetal and the one Cbz-group were removed by an exhaustive hydrogenolysis upon exposure to excess H_2_ and catalytic Pd-C to cleanly give the desired β-GAR (**1**β) as a single diastereomer after filtration through PTFE filter (Scheme 7). The ability to generate β-GAR (**1**β) by this method proved to be crucial to this stereospecific synthesis as exposure to relatively neutral solid supports like Celite^®^ led to complete anomerization (vide infra).

**Scheme 8:**
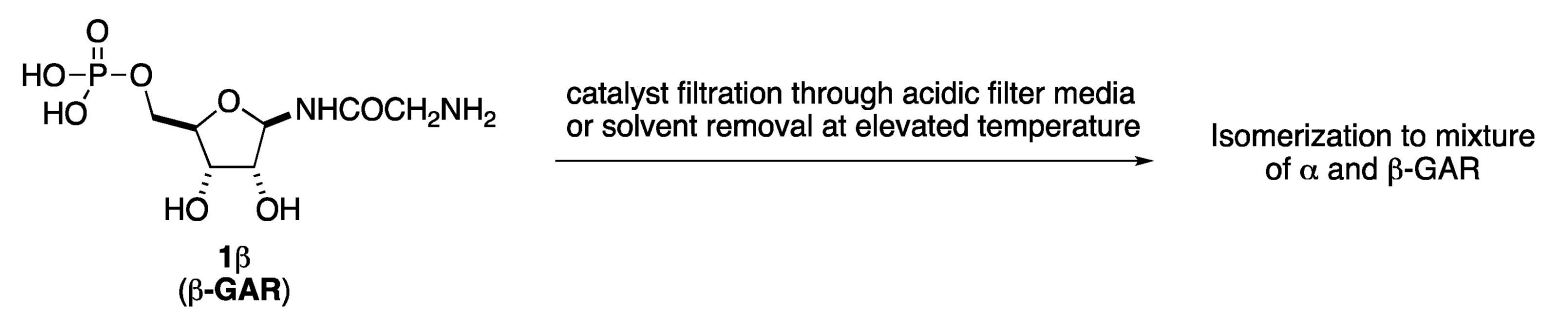
Isomerization under stressed conditions.

**Figure 1:**
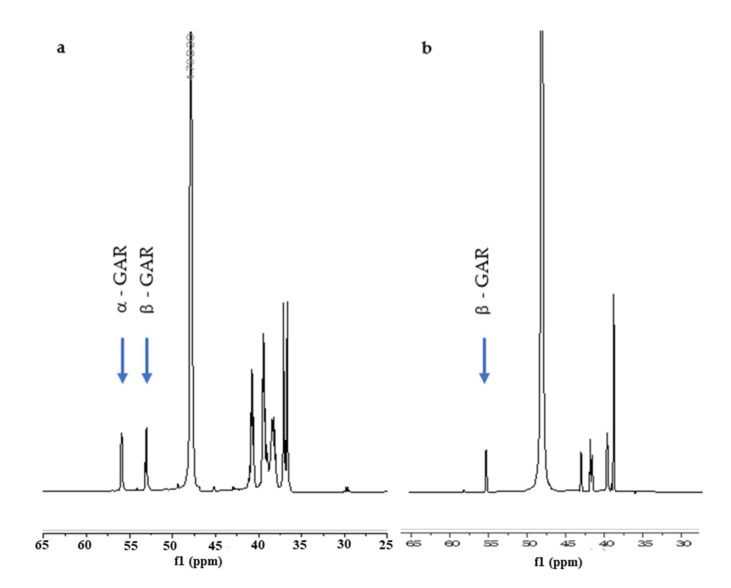
(**a**) ^1^H-NMR of a ~1:1 mixture of α- and β-GAR; (**b**) pure β-GAR.

**Figure 2:**
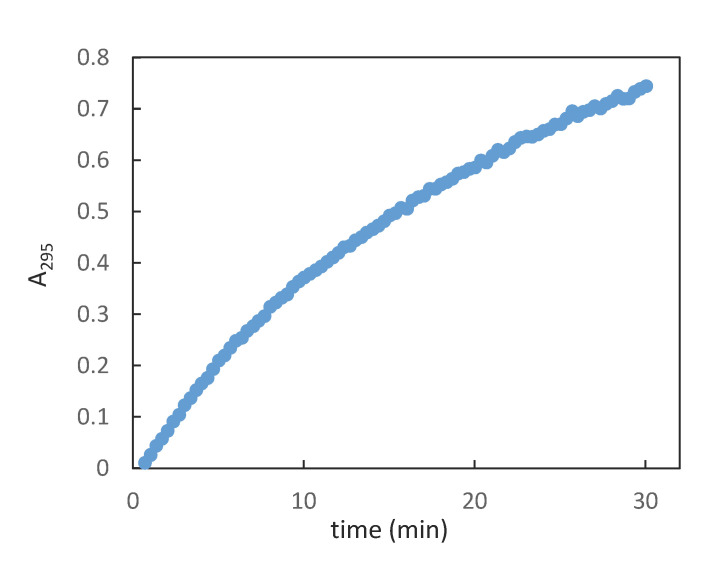
Increase in absorbance (295 nm) as a function of time; average of two experiments.

To our delight, the synthetic β-GAR (**1**β) generated by this procedure possessed a ^1^H- and ^13^C{^1^H}-NMR that match the data reported by Stubbe et al. [13] The spectral differences between the two anomers is readily apparent in the ^1^H-NMR, see Figure 1a (α/β-GAR (**1**α/β)) and Figure 1b (β-GAR (**1**β)). While not stable to acid, neutral aqueous solutions of β-GAR (**1**β) were stable for many days. A degree of isomerization (~45%) could be detected when the solvent (water) was removed at elevated temperature (40–50 °C, Scheme 8). The solid β-GAR (**1**β), obtained after lyophilization, was stable at −20 °C for over 6 months and was utilized as a substrate by GART as evidenced by an increase in A_295_ with time (Figure 2).

## 3. Conclusions

In conclusion, a stereoselective synthesis of β-GAR (**1**β) has been achieved, the nine-step synthesis provides access to diastereomerically pure β-GAR (**1**β) without the need for complex chromatography of the zwitterionic amino-phosphate. The synthesis was accomplished in 5% overall yield and provided material on the multi-mg scale. The material so produced was suitable for use in a fDDF/GART formyl-transfer assay.

## 4. Materials and Methods

### 4.1. General Methods

^1^H and ^13^C{^1^H} spectra were recorded on 270 and 600 MHz NMR spectrometers. Chemical shifts were reported relative to internal tetramethylsilane (δ 0.00 ppm) or CDCl_3_ (δ 7.26 ppm) or CD_3_OD (δ 4.89 ppm) for ^1^H-NMR and CDCl_3_ (δ 77.1 ppm) or CD_3_OD (δ 49.15 ppm) for ^13^C{^1^H}-NMR (see Supplementary Materials). Optical rotations were measured with a digital polarimeter in the solvent specified. Infrared (IR) spectra were obtained on a FT-IR spectrometer. Flash column chromatography was performed on ICN reagent 60 (60–200 mesh) silica gel. Analytical thin-layer chromatography was performed with precoated glass-backed plates (K6F 60 Å, F254) and visualized by quenching of fluorescence and by charring after treatment with *p*-anisaldehyde or phosphomolybdic acid or potassium permanganate stain. R*_f_* values were obtained by elution in the stated solvent ratios (*v*/*v*). Ether, tetrahydrofuran, methylene chloride, and triethylamine were dried by passing through activated alumina (8 × 14 mesh) column with nitrogen gas pressure. Commercial reagents were used without purification unless otherwise noted. Air and/or moisture-sensitive reactions were carried out under an atmosphere of argon/nitrogen using oven/flamed-dried glassware and standard syringe/septa techniques.

### 4.2. Experimental Procedures:

#### 4.2.1. 1-Azido-β-d-ribofuranosyl-5-acetate (**15**β)

2,3-*O*-Isopropyliden-5-*O*-acetyl-β-d-ribofuranosyl azide (**14**β) (257 mg, 1 mmol) was dissolved in 3:1 AcOH:H_2_O (6 mL) and refluxed for 5 h. The reaction was quenched by adding solid NaHCO_3_, diluted with DCM (50 mL) and the organic layer was washed with sat. NaHCO_3_ solution (5 mL × 3). The organic layer was dried over Na_2_SO_4_ and concentrated under reduced pressure. The crude product was purified by flash column chromatography by loading on sílica gel and eluting with 50% EtOAc/Hexane, the product containing fractions were concentrated to yield diol (**15**β) (169 mg, 0.78 mmol, 78%) as a clear oil. R*_f_* (70% EtOAc/Hexane) = 0.33; [α]_D_^24^ −279 (*c* 0.66, CH_2_Cl_2_); IR (neat) 3404, 2946, 2108, 1715, 1434, 1368, 1223 cm^−1^. ^1^H-NMR (500 MHz, CDCl_3_) δ 5.30 (s, 1 H), 4.42 (dd, *J* = 12, 3 Hz, 1 H), 4.20–4.29 (m, 2 H), 4.14–4.19 (m, 1 H), 3.97 (d, *J* = 4.5 Hz, 1 H), 2.93 (s, 1 H), 2.79 (s, 1 H), 2.13 (s, 3 H); ^13^C{^1^H}-NMR (125 MHz, CDCl_3_) δ 179.0, 94.8, 81.7, 75.6, 71.3, 64.1, 21.1; Mass Calculated for [C_7_H_11_N_3_O_5_] 217.0699, found 217.0702.

#### 4.2.2. 2,3-*O*-Benzyliden-5-*O*-acetyl-β-d-ribofuranosyl Azide (**16**β)

The starting material (**15**β) (1.1 g, 5.1 mmol) was dissolved in dry acetonitrile (12.5 mL) at 20 ± 5 °C under nitrogen. Camphor sulfonic acid (116 mg, 0.5 mmol) and benzaldehyde dimethyl acetal (0.84 mL, 5.6 mmol) were added, and the reaction mixture was stirred for an hour. The reaction was quenched by the addition of water (5 mL) and the product was extracted with EtOAc (3 × 20 mL). The combined organic layers were washed once with sat. NaHCO_3_ (10 mL) and once with brine (10 mL) and dried over Na_2_SO_4_. The solvent was removed under reduced pressure and the crude product was purified by flash column chromatography by loading on silica gel and eluting with 8% EtOAc/Hexane, concentration of product containing fractions afforded benzylidene azide (**16**β) (1.36 g, 4.46 mmol, 88%) as a clear viscous oil as the major isomer. R*_f_* (70% EtOAc/Hexane) = 0.8; [α]_D_^24^ −153 (*c* 0.12, CH_2_Cl_2_); IR (neat) 2949, 2110, 1740, 1452, 1402, 1371, 1314, 1219 cm^−1^. ^1^H-NMR (400 MHz, CDCl_3_) δ 7.45–7.52(m, 2 H), 7.36–7.45 (m, 3 H), 5.81 (s, 1 H), 5.71 (s, 1 H), 4.79 (d, *J* = 6 Hz, 2 H), 4.56–4.7 (m, 2 H), 4.17–4.33 (m, 2 H), 2.12 (s, 3 H); ^13^C{^1^H}-NMR (100 MHz, CDCl_3_) δ 170.9, 135.7, 130.4, 128.9, 127.2, 107.0, 96.8, 86.4, 85.1, 83.1, 64.0, 21.1; Mass Calculated for [C_14_H_15_N_3_O_5_Na^+^] 328.0904, found 328.0927.

#### 4.2.3. Benzyl(2-(2,3-*O*-benzyliden-5-*O*-acetyl-β-d-ribofuranosylamino)-2-oxoethyl)carbamate (**17**β)

Benzylidene azide (**16**β) (200 mg, 0.66 mmol), PPh_3_ (450 mg, 1.72 mmol), Cbz-Glycine (153 mg, 0.73 mmol), and Py_2_S_2_ (148 mg, 0.67 mmol) were added to a round bottom flask. The flask was sealed with a septum and placed in an ice bath. Under nitrogen, dry toluene (1.65 mL) was added to the flask with stirring. The solution was stirred overnight. The reaction was quenched by adding water. The product was extracted with EtOAc, dried over Na_2_SO_4_, and concentrated under reduced pressure. The crude product was passed through a silica gel plug by eluting with 55% EtOAc/Hexane. The fractions containing product were concentrated to yield the 5-*O*-acetyl-Cbz-glycinamide along with some by-product. R*_f_* (70% EtOAc/Hexane) = 0.4; [α]_D_^24^ −1.7 (*c* 0.26, CH_2_Cl_2_); IR (neat) 3419, 3349, 3052, 2922, 2357, 2337, 1736, 1703, 1681, 1539, 1505, 1455, 1437, 1267, 1217 cm^−1^. HRMS Calculated for [C_24_H_26_N_2_O_8_Na^+^] 493.1581, found 493.1592.

The 5-*O*-acetyl-Cbz-glycinamide obtained from the previous step was dissolved in MeOH (2.5 mL). NaOMe in MeOH (0.5 N, 1.25 mL) was added, and the mixture was stirred at 0 °C for 30 min. MeOH was removed by evaporation and the product was extracted in diethyl ether. The organic layer was dried over Na_2_SO_4_ and concentrated under reduced pressure. The β-anomer was separated by column chromatography by loading on silica gel and eluted with 80% EtOAc/Hexane. The pure β-anomer (**17**β) containing fractions were combined and concentrated to give a white solid, (76 mg, 0.18 mmol, 27% beta-anomer only). R*_f_* (70% EtOAc/Hexane) = 0.2; [α]_D_^24^ −1.7 (*c* 0.26, CH_2_Cl_2_); IR (neat) 2357, 2336, 1679, 1651, 1549, 1504, 1453, 1405 cm^−1^. ^1^H-NMR (400 MHz, CDCl_3_/CD_3_OD) δ 7.35–7.42 (m, 2 H), 7.16–7.31 (m, 8 H), 5.80 (s, 1 H), 5.69 (s, 1 H), 4.98 (s, 2 H), 4.76 (d, *J* = 6.4 Hz, 1 H), 4.54 (d, *J* = 6.0 Hz, 1 H), 4.29 (bs, 2 H), 3.55–3.77 (m, 6 H); ^13^C{^1^H}-NMR (100 MHz, CDCl_3_) δ 169.7, 157.3, 136.3, 135.9, 130.0, 128.9, 128.6, 128.5, 128.3, 128.1, 127.0, 106.5, 86.8, 86.4, 86.2, 83.1, 67.2, 62.8, 44.4. HRMS Calculated for [C_22_H_25_N_2_O_7_^+^] 429.1656, found 429.1645.

#### 4.2.4. *N*-Cbz-Dibenzyl-β-glycinamide Ribonucleotide (**18**β)

2,3-*O*-Benzylidene-1-*N*-(benzyloxycarbonylglycyl)-d-ribofuranosylamine (**17**β) (108 mg, 0.25 mmol) was dissolved in dry DCM (2.5 mL). A solution of dibenzyl *N*,*N*-diisopropylphosphoramidite (0.33 mL, 348 mg, 1.01 mmol) in dry DCM (3.75 mL), followed by tetrazole (2.2 mL, 1 mmol, 0.45 M in CH_3_CN) was added and the mixture was stirred at 20 ± 5 °C for 1 h. The reaction mixture was cooled to 0 °C, H_2_O_2_ (0.65 mL, 35% in H_2_O) was added and stirring was continued at 0 °C for 45 min. Upon complete consumption of the intermediate the reaction was quenched by adding sat. Na_2_SO_3_ dropwise with stirring over 10 min. The reaction mixture was extracted with EtOAc (10 mL × 2). The combined organics were washed once each with sat. NaHCO_3_ and brine and dried over Na_2_SO_4_. After solvent removal, the crude product was purified by flash column chromatography by loading on silica gel and eluting with 45–50% EtOAc/hexanes, the product containing fractions were combined and concentrated to obtain the phosphate (**18**β) as a clear viscous oil (144 mg, 1 mmol, 83%). R*_f_* (70% EtOAc/Hexane) = 0.8; [α]_D_^24^ −8.4 (*c* 0.09, CH_2_Cl_2_); IR (neat) 3305, 3035, 2927, 1723, 1693, 1498, 1455, 1437, 1393, 1264, 1216 cm^−1^; ^1^H-NMR (400 MHz, CDCl_3_) δ 7.67 (dd, *J* = 12, 7.2 Hz, 2 H), 7.23–7.58 (m, 18 H), 5.87 (d, *J* = 7.2 Hz, 1 H) 5.79 (s, 1 H), 4.93–5.20 (m, 6 H), 4.48–4.59 (m, 2 H), 4.42 (s, 1 H), 3.94–4.13 (m, 2 H), 3.75–3.84 (m, 2 H); ^13^C{^1^H}-NMR (100 MHz, CDCl_3_) δ 169.5, 156.8, 136.7, 135.9, 135.6, 133.4, 132.5, 132.4, 132.3 (2 Cs), 130.3, 129.1 (2 Cs), 128.9 (2 Cs), 128.8 (2 Cs), 128.6 (2 Cs), 128.5, 127.2, 107.1, 88.9, 86.8, 84.7, 82.3, 70.4 (2 Cs), 68.8, 67.3, 44.8; HRMS Calculated for [C_36_H_37_N_2_O_10_P] 711.2078, found 711.2054.

#### 4.2.5. β-Glycinamide Ribonucleotide (**1**β)

2,3-*O*-Benzylidene-1-*N*-(benzyloxycarbonylglycyl)-d-ribofuranosylamine-5-dibenzylphosphate (**18**β) (16 mg, 0.023 mmol) was dissolved in MeOH (0.15 mL) and water (0.5 mL). Pd-C was added, and the reaction mixture was stirred under hydrogen overnight when global deprotection went to completion. After filtering through a PTFE syringe filter to remove Pd-C, the filtrate was lyophilized to give β-GAR (**1**β) (5.7 mg, 0.02 mmol, 86%) as flaky solids. R*_f_* (70% EtOAc/Hexane) = 0; [α]_D_^24^ +5 (*c* 0.7, H_2_O with 0.25 mM Tris buffer); ^1^H-NMR (500 MHz, D_2_O) δ 5.32 (d, *J* = 5.5 Hz, 1 H), 4.06–4.12 (m, 1 H), 3.92–3.99 (m, 2 H), 3.71- 3.78 (m, 2 H), 3.67 (s, 2 H). ^13^C{^1^H}-NMR (100 MHz, D_2_O) δ 168.3, 83.7, 83.0, 74.1, 70.1, 64.7, 40.8. HRMS Calculated for [C_7_H_15_N_2_O_8_PH^+^] 287.0639, found 287.0645.

#### 4.2.6. Isomerization of β-GAR

When the crude reaction mixture of β-GAR (**1**β) prepared from the above protocol was filtered through Celite^®^, a 1:1 mixture of anomers α/β-Gar (**1**α/β) was formed. The product β-GAR (**1**β) was isolated as a colorless, syrupy liquid. ^1^H-NMR (400 MHz, D_2_O) δ 5.65 (d, *J* = 4 Hz), 5.36 (d, *J* = 4.2 Hz), 4.13–4.18 (m), 3.96–4.04 (m), 3.75–3.92 (m), 3.75 (s), 3.72 (s). ^13^C{^1^H}-NMR (125 MHz, D_2_O) δ 168.18, 168.16, 83.6, 82.8 (d, *J* = 9 Hz), 81.0 (d, *J* = 8 Hz), 80.5, 74.0, 70.8, 70.7, 70.2, 65.0 (d, *J* = 5 Hz), 64.8 (d, *J* = 5 Hz), 40.8, 40.7. IR (neat): ν 3215.7, 2929.6, 1674.0, 1536.2, 1434.4, 1129.8, 1027.8 cm^−1^. HRMS Calculated for [C_7_H_15_N_2_O_8_PH^+^] 287.0639, found 287.0645. [α]_D_^24^: −21.1 (*c* 0.10, H_2_O).

#### 4.2.7. GART Activity

His-tagged *E. coli* GART (*purN* gene) was expressed from pCA24N obtained from ASKA [29], with 0.5 mM IPTG (f.c.) added at 0.6–0.8 A_600_, then grown at 30 °C for 4 h and harvested at 6000× *g* for 10 min. Cell pellets were sonicated in equilibration buffer (50 mM HEPES, pH 8, 500 mM NaCl, 10 mM imidazole, 5 mM 2-mercaptoethanol, BME) and treated with DNase I and lysozyme on ice for 1 h, followed by centrifugation at 14,000× *g* to clear the lysate. The supernatant was loaded onto a 10 mL HisTrap FF column (GE Healthcare) with equilibration buffer and elution buffer containing 50 mM HEPES, pH 8, 500 mM NaCl, 500 mM imidazole, 1 μL/mL of PMSF, 5 mM BME, and half of a protease inhibitor cocktail tablet (Roche) over a linear gradient of 10–500 mM imidazole. Elution fractions were dialyzed overnight and concentrated to ~5 mL with 10 kDa MWCO Vivaspin 6 (Vivaproducts) concentrators at 7000× *g*. Concentrated protein was stored in 50 mM HEPES, pH 8, 200 mM NaCl, 5 mM BME at −80 °C. Enzymatic assays were carried out essentially as described by monitoring A_295_ at 20 ± 1 °C in 50 mM HEPES, pH 8, 20 mM NaCl buffer, 1 mM β-GAR, 1 mM fDDF, and initiated with 25 nM enzyme [30]. 

## Data Availability

Data is contained within the article or Supplementary Materials.

## Supplementary Materials

The following are available online at https://www.mdpi.com/article/10.3390/molecules27082528/s1, Copies of ^1^H- and ^13^C{^1^H}-NMR spectra for all new compounds.

## Sample Availability

Samples of the compounds are available from the authors.
