# Supplementary figures and images for: Stereoselective Synthesis of β-Glycinamide Ribonucleotide
Source: Molecules. 2022 Apr 14;27(8):2528. doi: 10.3390/molecules27082528 (PMC9024515; doi:10.3390/molecules27082528)

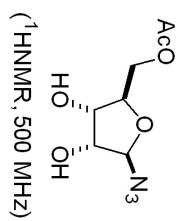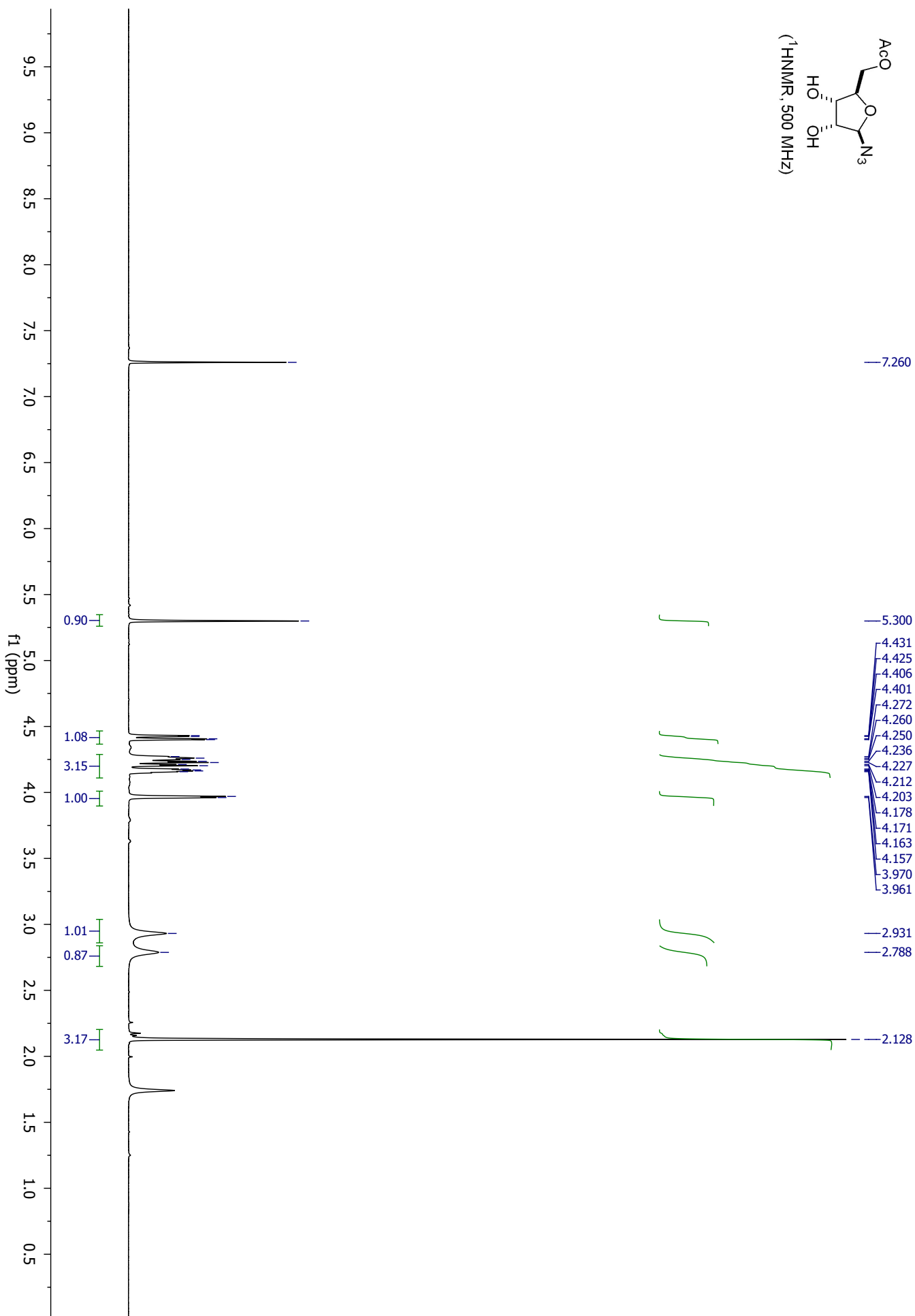

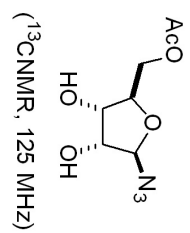

—179.0

—94.8

81.7  
 77.6  
 77.4  
 77.1  
 75.6  
 71.5

—64.1

—21.1

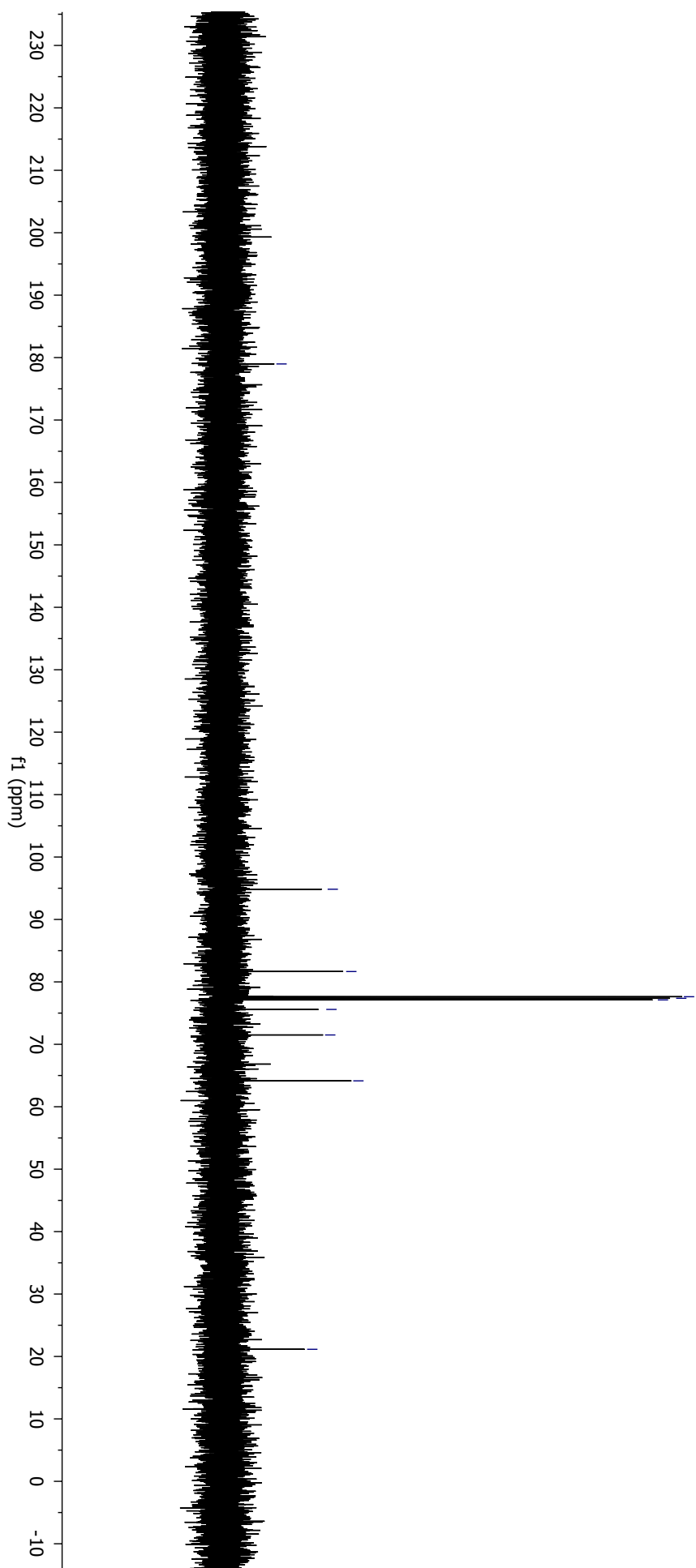

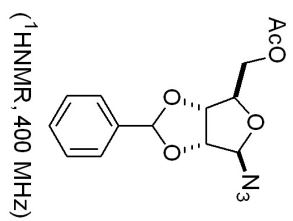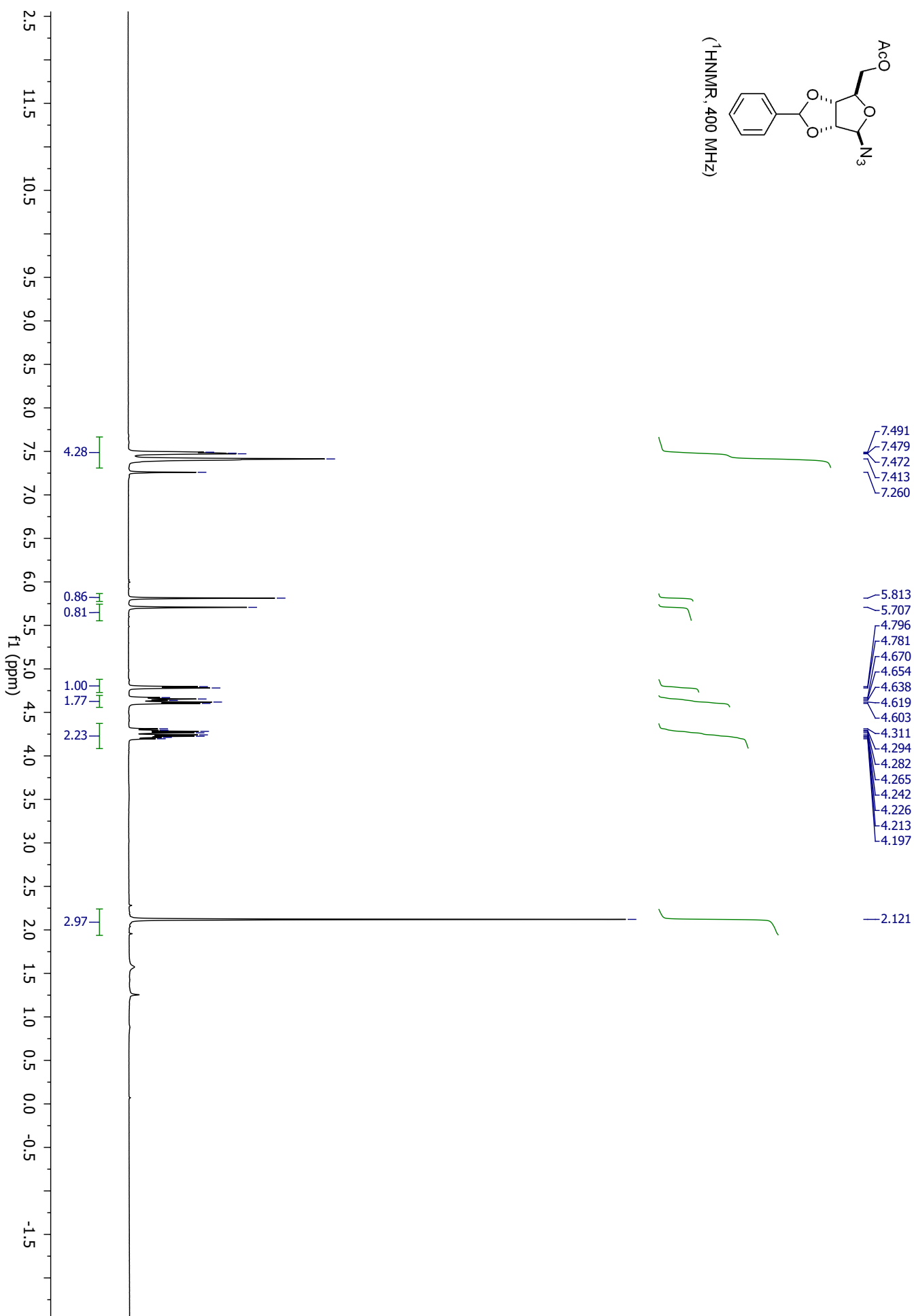

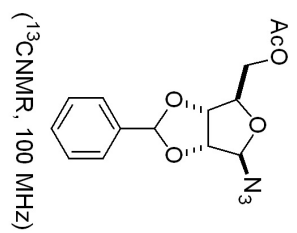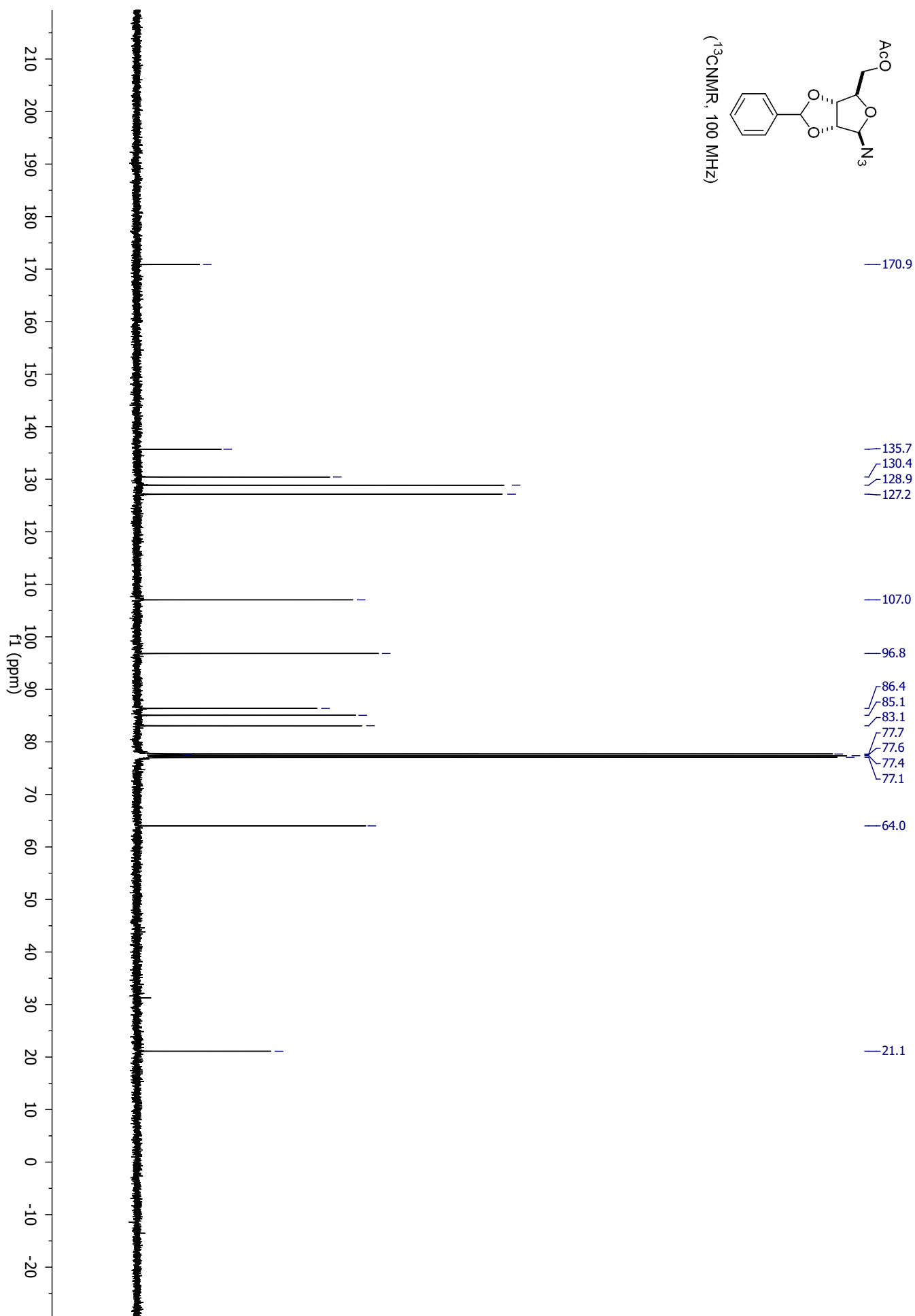

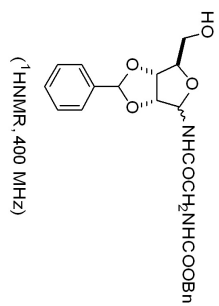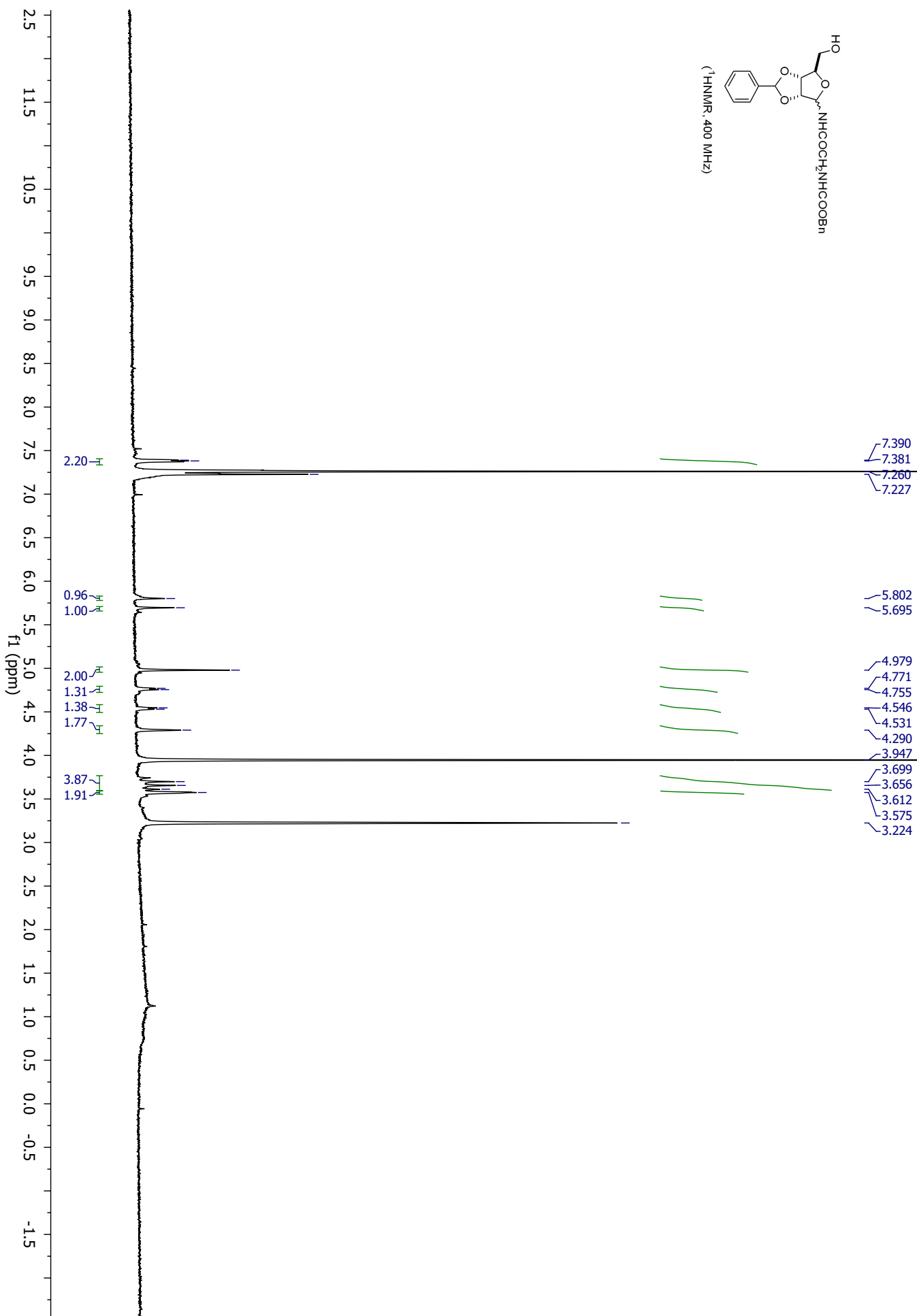

DR-IV-13-run2-1H  
 NU STANDARD 1H OBSERVE  
 DR-IV-13-run2-1H

5.794  
 5.686  
 5.630

4.971  
 4.939  
 4.762  
 4.747  
 4.538  
 4.523

4.282

3.981

3.732  
 3.689  
 3.647  
 3.603  
 3.572  
 3.566  
 3.519  
 3.215

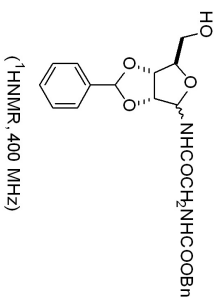

(<sup>1</sup>H NMR, 400 MHz)

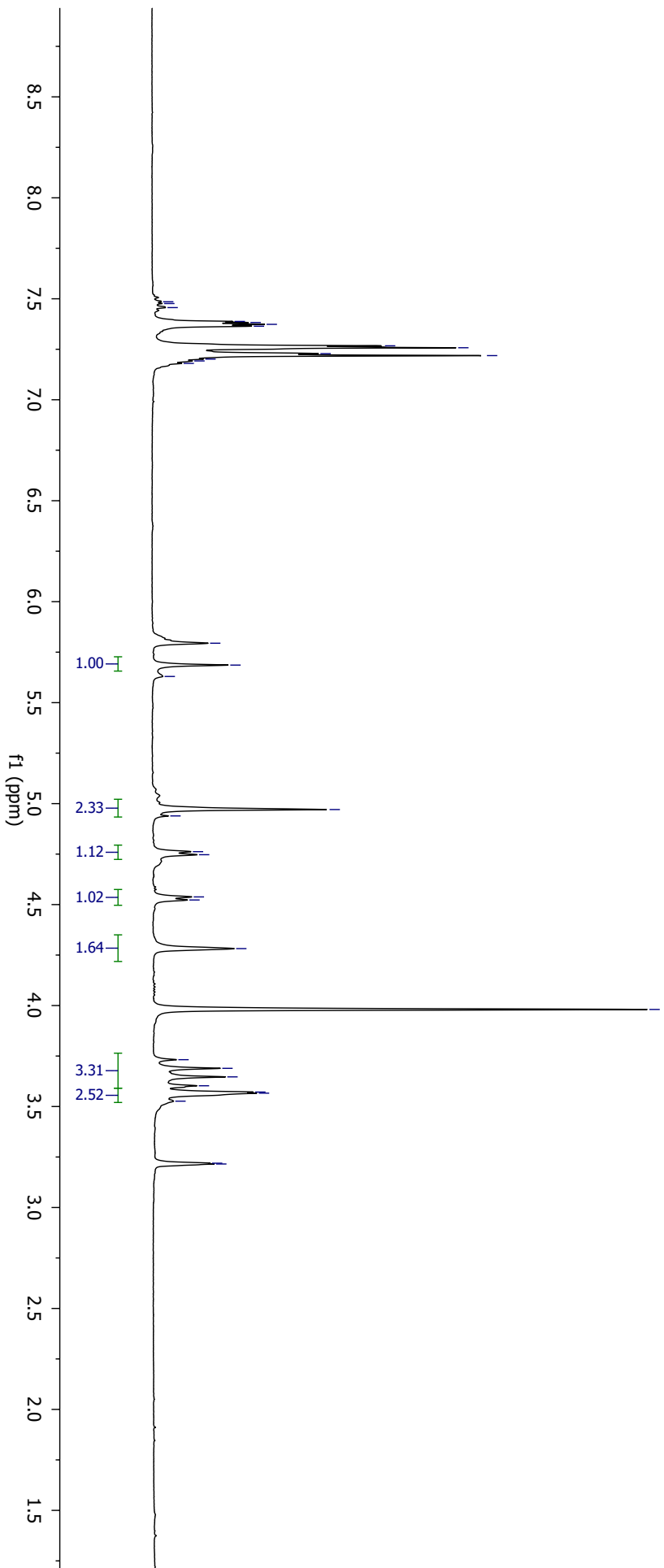

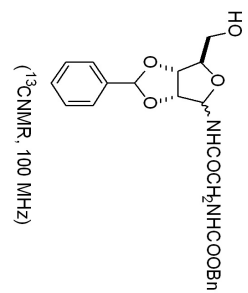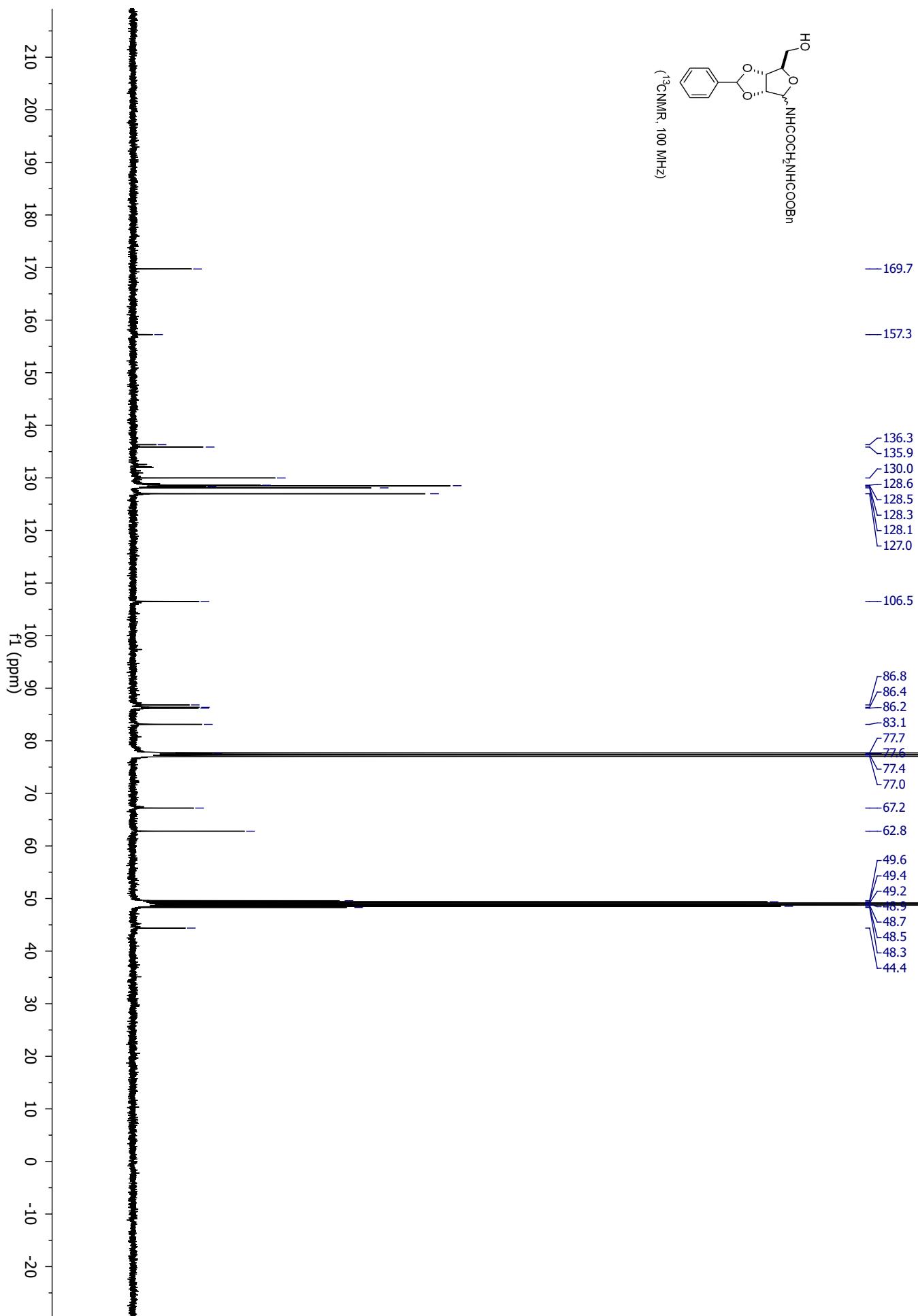

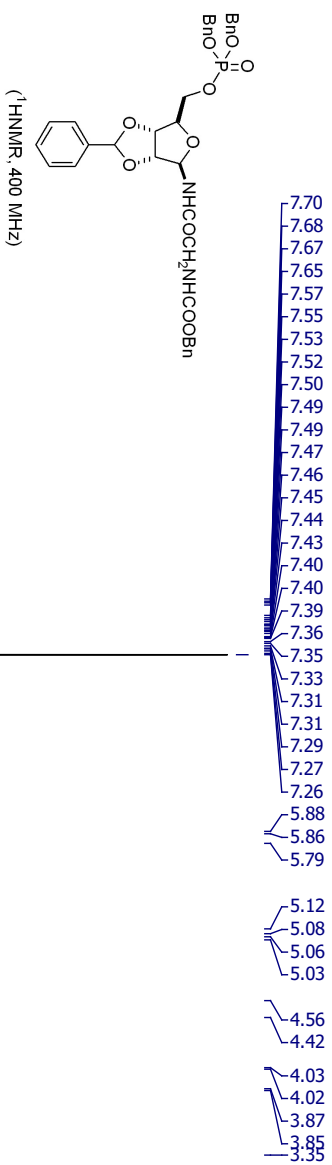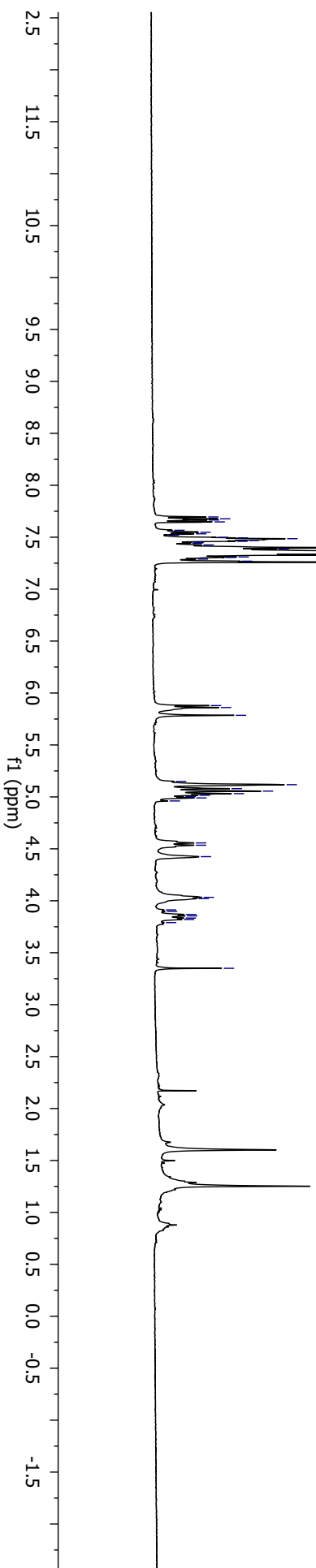

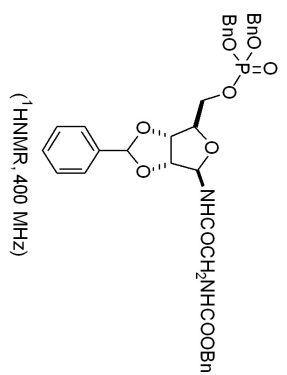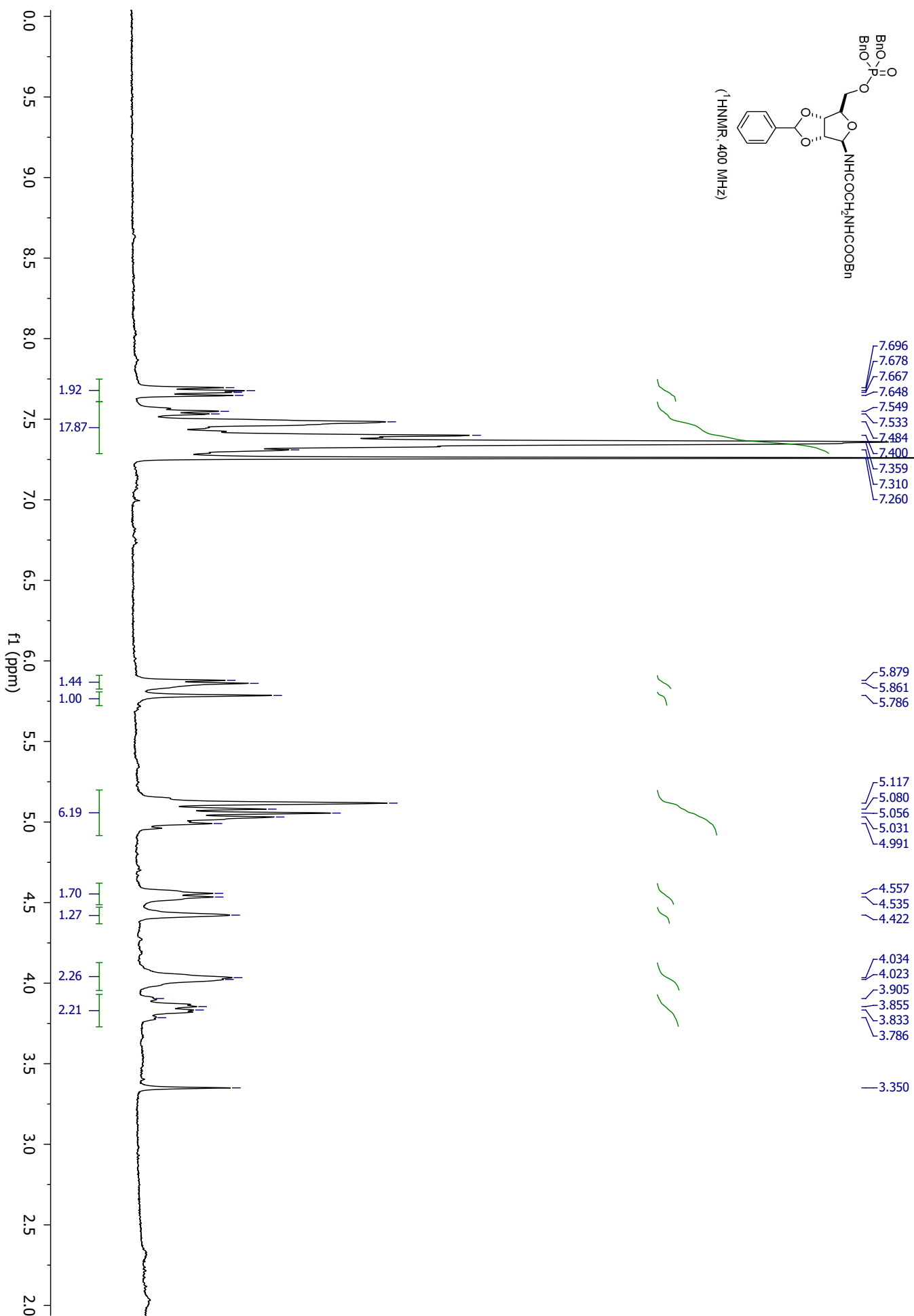

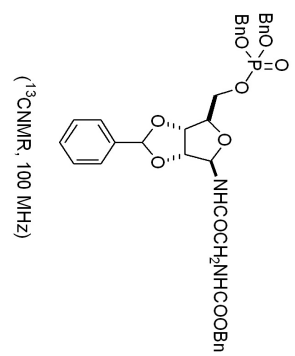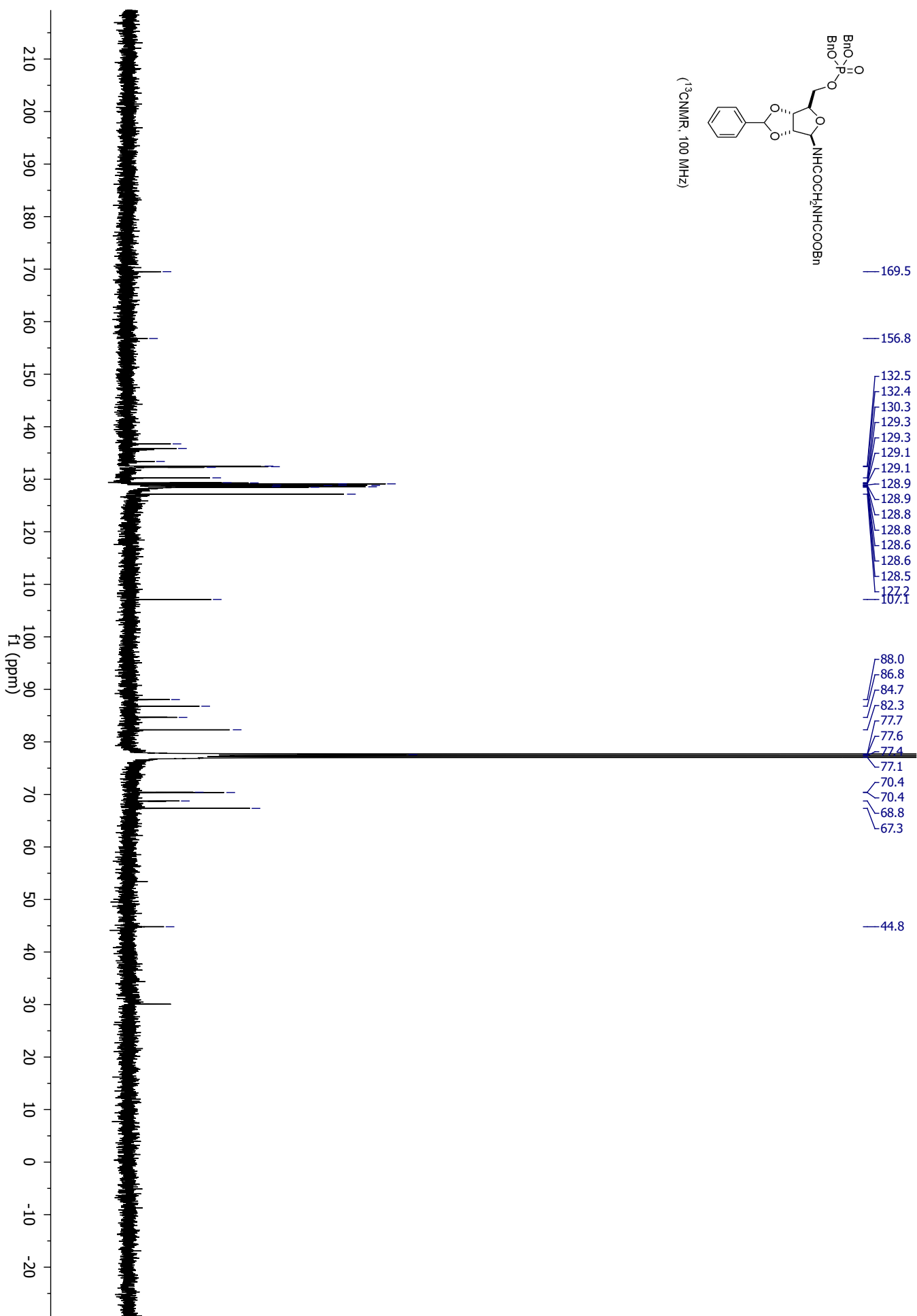

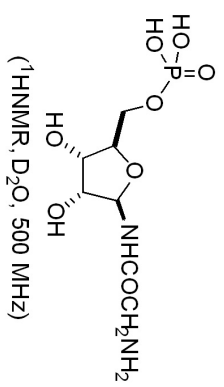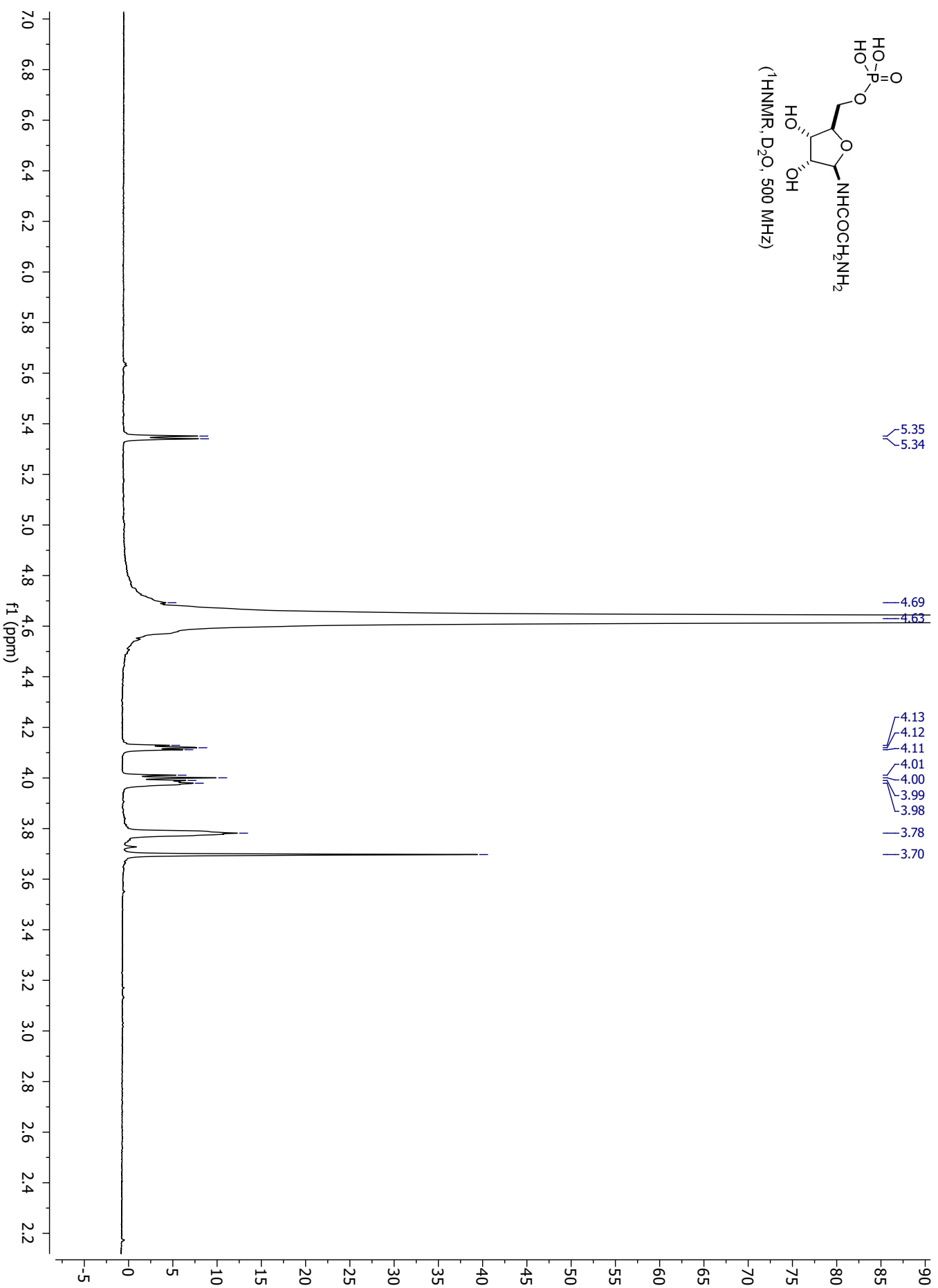

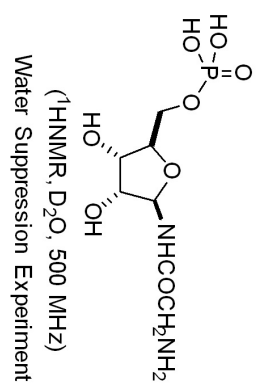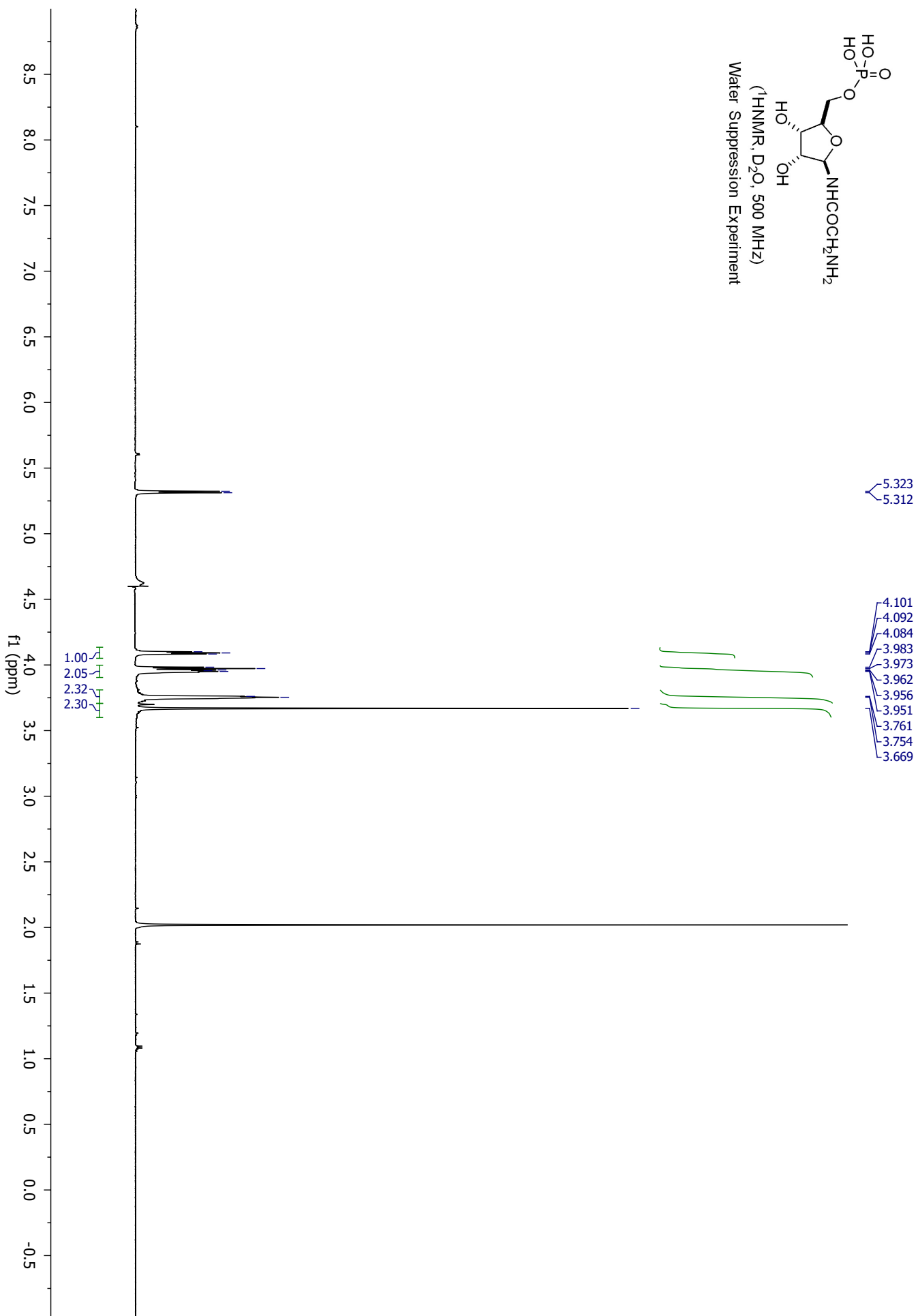

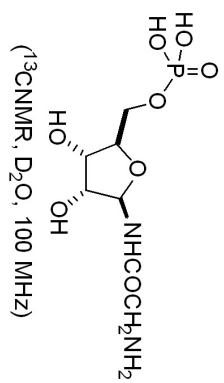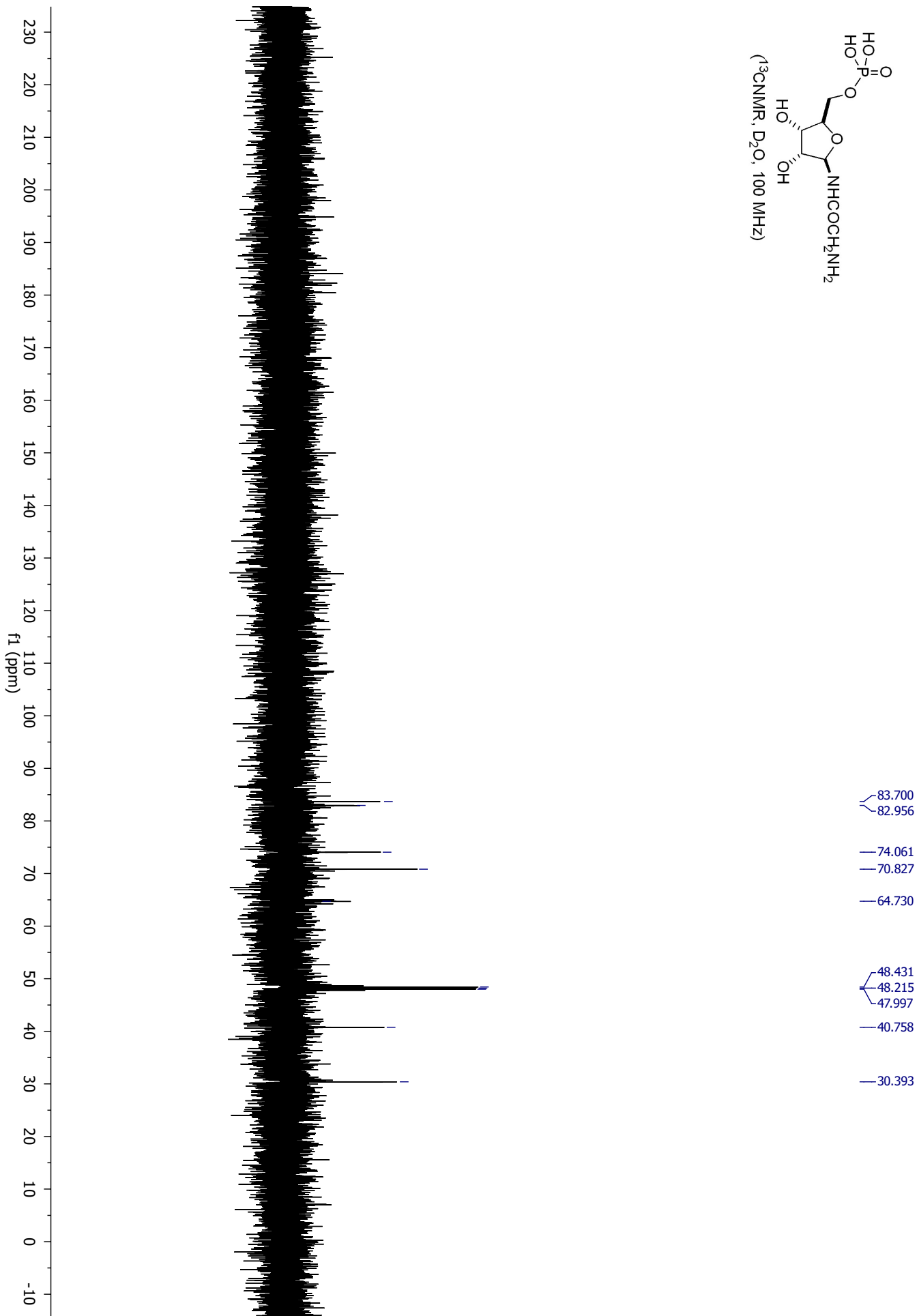

Supplement: Supplementary file 1 [file molecules-27-02528-s001.zip › molecules-1607152-supplementary.pdf]
